# Blood Ketone Bodies and Breath Acetone Analysis and Their Correlations in Type 2 Diabetes Mellitus

**DOI:** 10.3390/diagnostics9040224

**Published:** 2019-12-17

**Authors:** Valentine Saasa, Mervyn Beukes, Yolandy Lemmer, Bonex Mwakikunga

**Affiliations:** 1 DSI/CSIR Centre for Nanostructures and Advanced Materials, P.O. Box 3951, Pretoria 0001, South Africa; 2 Department of Biochemistry, Genetics and Microbiology, University of Pretoria, Pretoria 0001, South Africa; 3 Department of Biochemistry, Stellenbosch University, Cape Town 7600, South Africa; mervynbeukes@sun.ac.za; 4 Next Generation Health, Division 1, CSIR, P.O. Box 3951 Pretoria, South Africa; ylemmer@csir.co.za; 5 Department of Physics, Tshwane University of Technology, P.O. Box x680, Pretoria 0001, South Africa

**Keywords:** diabetes mellitus, ketone bodies, human breath, acetone, beta-hydroxybutyrate, acetoacetate, gas chromatography-mass spectrometry (GC-MS)

## Abstract

Analysis of volatile organic compounds in the breath for disease detection and monitoring has gained momentum and clinical significance due to its rapid test results and non-invasiveness, especially for diabetes mellitus (DM). Studies have suggested that breath gases, including acetone, may be related to simultaneous blood glucose (BG) and blood ketone levels in adults with types 2 and 1 diabetes. Detecting altered concentrations of ketones in the breath, blood and urine may be crucial for the diagnosis and monitoring of diabetes mellitus. This study assesses the efficacy of a simple breath test as a non-invasive means of diabetes monitoring in adults with type 2 diabetes mellitus. Human breath samples were collected in Tedlar™ bags and analyzed by headspace solid-phase microextraction and gas chromatography-mass spectrometry (HS-SPME/GC-MS). The measurements were compared with capillary BG and blood ketone levels (β-hydroxybutyrate and acetoacetate) taken at the same time on a single visit to a routine hospital clinic in 30 subjects with type 2 diabetes and 28 control volunteers. Ketone bodies of diabetic subjects showed a significant increase when compared to the control subjects; however, the ketone levels were was controlled in both diabetic and non-diabetic volunteers. Worthy of note, a statistically significant relationship was found between breath acetone and blood acetoacetate (*R* = 0.89) and between breath acetone and β-hydroxybutyrate (*R* = 0.82).

## 1. Introduction

Human biological samples such as breath, blood and urine contain several volatile organic compounds (VOCs). These VOCs are associated with specific metabolic pathways, and are useful as biomarkers reflecting the disease and physiological state of a human that cause changes in their metabolism [1]. Particularly, analysis of breath has been receiving more attention because of its potential as a non-invasive method for disease diagnosis and metabolic status monitoring [2]. Among thousands of VOCs, acetone is the second to highest in abundance in normal human breath gases, which has been extensively studied as a breath biomarker of diabetes or as a high abundant breath VOC in various physiological cases since the 1950s [1,3]. The studies which showed a strong link between breath acetone and plasma glucose are mostly for type 1 diabetes, but no such observation has been obtained so far from adequately controlled type 2 diabetes mellitus patients [4,5,6,7]. 

In addition, breath acetone concentration (BrAce) is also well understood to be a non-invasive measure of ketosis. Ketosis is a metabolic state characterized by the elevation of ketone bodies in the blood. Healthy individuals on standard mixed diets (i.e., moderate to high carbohydrate content) have basal ketosis, while individuals with uncontrolled diabetes have extremely elevated ketosis, which could lead to ketoacidosis. In all cases, ketosis describes the quantity of circulating ketone bodies [8].

**Figure 1:**
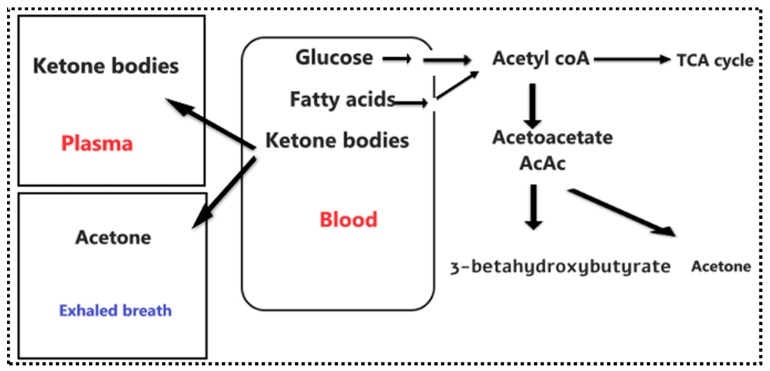
Schematic diagram of the formation of ketone bodies (acetoacetate, beta-hydroxybutyrate, and acetone) which takes place in the mitochondrial matrix of the liver cell. The acetyl-coenzyme A (acetyl-CoA) can be metabolized through the tricarboxylic acid (TCA) cycle, or can undergo ketogenesis. The three ketone bodies travel through the blood and acetone is also expelled the breath. (Red is for ketones in the blood and blue is for ketones in the human breath).

Ketone bodies are produced as a by-product of the fat metabolism process [9]. When the liver metabolizes circulating free fatty acids, these acids are transformed into acetyl-coenzyme A (acetyl-CoA), a molecule used in the production of energy. The acetyl-CoA linked to the tricarboxylic acid cycle (TCA cycle) is produced from glucose and fatty acids (Figure 1). Before acetyl CoA enters the TCA cycle, it first condenses with oxaloacetate, and when the glucose availability is reduced in the liver due to: fasting, a low-carbohydrate diet, insulin deficiency or insulin resistance caused by diabetes, the production of acetyl CoA from fatty acids increases. The resultant excess acetyl CoA is diverted to the production of ketone bodies. As a result, blood and breath acetone concentration increase [10]. Indeed, elevated acetone concentrations within exhaled breath have been observed in diabetes mellitus patients [6,11,12,13,14].

The world prevalence of diabetes mellitus among adults (aged 20–79 years) in 2010 was 6.4%, affecting 285 million adults, and is estimated to increase to 7.7%, affecting 439 million adults by 2030. Between 2010 and 2030, there will be a 69% increase in the numbers of adults with diabetes in developing countries, and a 20% increase in developed countries [15]. South Africa, as one of the developing countries, has half a million people (about 6% of the population) suffering from diabetes mellitus, the majority having type 2 diabetes mellitus [16,17]. The major abnormalities in type 2 diabetes mellitus include dyslipidemia, insulin resistance, hyperglycemia and ketoacidosis, which usually are not taken into consideration in type 2 diabetes mellitus [18]. Additionally, the diagnosis and monitoring of blood glucose and ketone bodies for diabetic patients involves the use of blood tests. Usually, this is done by drawing blood from a patient for analysis and using a glucose measuring device such as a glucometer. These methods are painful, invasive and time-consuming [19]. A great opportunity lies in the use of breath acetone for diagnosis and monitoring of the disease. This might suggest the potential to develop breath gas analysis to provide an alternative to blood testing for glucose and ketone measurement. This could offer patients a non-invasive, pain free and point of care solution to their daily lives.

Studies have suggested that breath gases, including acetone, may be related to simultaneous blood glucose (BG) and blood ketone levels in type 1 diabetes [20,21,22]. However, from our knowledge based on literature, there has not been a study on the analysis or correlation of ketone bodies and breath acetone on type 2 diabetes mellitus. In this study, we assessed the relationship between blood ketone bodies and breath acetone, along with the blood glucose and breath acetone of type 2 diabetes mellitus, and determined whether breath acetone could be used as a biomarker for diabetes mellitus. The aim of the present study is to establish the relationship between breath acetone and plasma ketones of non-diabetes mellitus volunteers and type 2 diabetes mellitus patients.

While breath acetone and blood ketone bodies have been measured in relatively large cohorts of diabetes mellitus patients, most breath and blood ketone measurements have been carried out on only type 1 diabetes mellitus. For example, Blaikie et al. [20] measured breath acetone and blood ketone bodies in children with type 1 diabetes mellitus, Minh et al. [23] showed that concentrations of two separate groups of acetone, methyl nitrate, ethanol and methylbenzene and acetone, methanol, propane and 2-pentyl nitrate were able to demonstrate that blood glucose can be measured simultaneously with breath acetone in both healthy adults with type 1 diabetes mellitus. A previous study from the same group demonstrated a correlation between exhaled methyl nitrate and blood glucose in a cohort of young people with type 1 diabetes mellitus [24]. Many more studies on breath acetone, blood ketone and blood glucose have been reported [7,25,26]. Nevertheless, the urge to look for the non-invasive monitoring of both blood glucose and ketone bodies should not be limited to only type 1 diabetes mellitus, as type 2 diabetic patients can also suffer from ketoacidosis, and this therefore also requires daily monitoring of their blood glucose non-invasively.

The aim of this study is to assess the efficacy of a simple breath test as a non-invasive means of diabetes monitoring in type 2 diabetes mellitus patients. Human breath samples were collected in Tedlar™ bags and analyzed by HS-SPME/GC-MS. The measurements were compared with capillary fasting blood glucose (BG) and ketone levels taken at the same time on a single visit to a routine hospital clinic in 30 subjects with type 2 diabetes mellitus and 28 control volunteers. A statistically significant relationship was found between breath acetone and blood acetoacetate (*R* = 0.897), and between breath acetone and β-hydroxybutyrate (*R* = 0.821).

## 2. Materials and Methods

### 2.1. Study Population

A total of thirty (30) diabetes mellitus and twenty-eight (28) controls participants aged between 18 to 60 years were recruited for this study based on the prevalence of diabetes in this locality. The patients were all non-smokers. Inclusion criteria include being diabetic, while an exclusion criterion involved being non-diabetic and having any other chronic illness. The fasting blood was used. A routine glucose test was also performed for participants to confirm the diabetic state. Informed consent was obtained from participants as well as ethical clearance (Ref: 118/2015, 21 November 2016) from the ethics committee of the Council for Scientific and Industrial Research (CSIR).

### 2.2. Collection of Samples

Blood samples were collected by standard vein puncture into the plain tube in the morning before a meal (after 8 h since the last meal). The blood was centrifuged at 3000 rpm for 10 min. The serum was separated into a separate tube and kept in −80 °C freezer until analysis. The breath samples were also collected simultaneously with blood samples using the Tedlar™ bags via a two-way non-rebreathing valve and analyzed immediately using HS-SPME/GC-MS. The participants were asked to inhale moderately and then to exhale as much as possible into a 3-lTedlar™ bag. Tedlar™ bags were first flushed with with pure nitrogen gas prior to the collection of breath samples.

### 2.3. Biochemical Analysis in the Blood

Ketone bodies (acetoacetate and beta-hydroxybutyrate), were analyzed using Abcam’s Acetoacetate (ab180875) and Beta-hydroxybutyrate (ab83390), respectively. The Acetoacetate kits provide a sensitive method to quantitate endogenous levels of AcAc in human blood. In this non-enzymatic assay, AcAc reacts with a substrate to generate a colored product that can be measured at 550 nm [27]. The reaction is specific for AcAc, and does not detect beta-hydroxybutyrate. The beta-hydroxybutyrate kits utilize beta HB dehydrogenase to generate a product that reacts with the colorimetric probe with an absorbance peak of 450 nm [28].

Serum from 30 diabetic and 28 non-diabetic mellitus patients were analyzed using Abcam’s glucose assay kit to quantify the amount of glucose in the blood. In this assay, glucose oxidase specifically oxidizes glucose to generate a product which reacts with a dye to generate color (570 nm). The generated color is proportional to the glucose amount.

### 2.4. Breath Acetone Analysis Using HS-SPME/GC-MS

**Figure 2:**
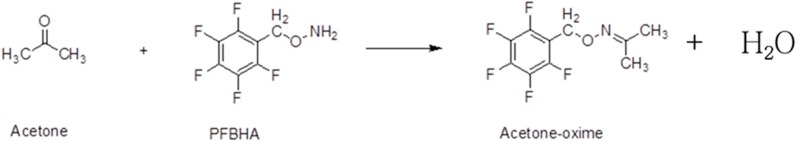
Schematic representation of the reaction between breath acetone and the derivatizing agent (PFBHA) reacting on the solid-phase microextraction (SPME) fiber.

In this study, we used the HS-SPME/GC-MS to quantify the breath acetone in diabetic and non-diabetic mellitus patients. The method is very simple, fast and sensitive for the optimization and accurate quantification of acetone in human breath. Acetone gas standards in the concentration range of 0.073, 0.59, 1.66, 3.32 and 6.64 ppmv were prepared. A solid-phase microextraction (SPME) fiber pre-coated with 20 mg/mL of *O*-2,3,4,5,6-(pentafluorobenzyl) hydroxylamine hydrochloride (PFBHA) was exposed inside the 3 L Tedlar™ bag at 40 °C containing the acetone standards and human breath for 10 min. Acetone present in the breath samples underwent on-fiber SPME derivatization to form the stable oxime (Figure 2). 

An Agilent Technologies model 6890N Gas Chromatography interfaced with Agilent Technologies model 6890N Mass Selective Detector was used for analysis. A 30 m × 0.25 mm with 0.25 µm RXi^®^-5 SilMS (Restek, Bellefonte, Pennsylvania, USA) was used as the analytical column. The injection port temperature which was also the temperature for thermal desorption of VOC was 250 °C, and the desorption time was 2 min. The GC split valve was set to open after the 2 min desorption Btime. The GC injector liner was a quartz SPME liner with an internal diameter of 0.75 mm (Supelco Inc., Bellefonte, PA, USA). The column temperature was regulated through the program, an initial temperature of 60 °C, was increased to 150 °C at 10 °C /min, and then increased to 300 °C (and held for 1 min). Total ion current was monitored using the electron-impact ionization mode (70 EV). Quantification was performed using characteristic mass. The peak at *m*/*z* 181 was used for the quantification of the acetone-PFBHA derivative.

### 2.5. Statistical Analysis

Statistical Software Package for Social Sciences (SPSS) 26 was used for data processing. Results are expressed in mean ± standard deviation (SD) or median (range). The correlation studies were investigated using Pearson and Spearman’s rank correlations. Linear models were then fitted with the breath acetone concentration as response variables. The data for qualitative comparison was analyzed by using Levene’s *t*-test. A *p*-value < 0.05 was considered statistically significant.

## 3. Results

### 3.1. Biochemical Analysis

**Figure 3:**
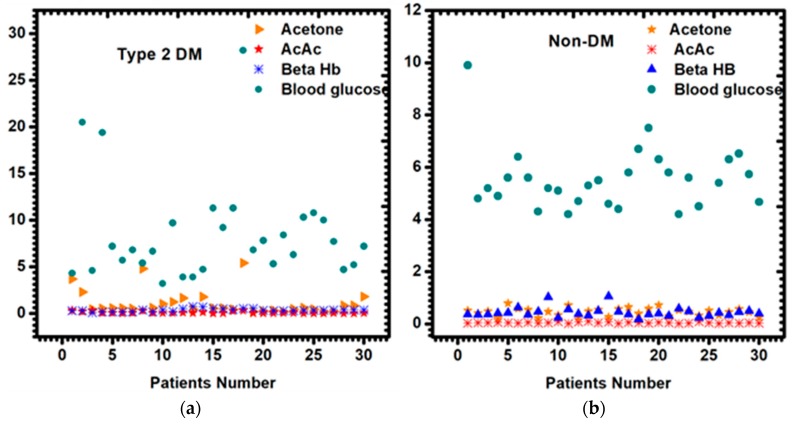
Scatter plot for plasma blood glucose, acetoacetate, beta-hydroxybutyrate and breath acetone in (**a**) type 2 diabetic and (**b**) non-diabetic mellitus patients.

Assessment of ketone bodies is an important practice more especially in clinical institutions to check and monitor for diabetic ketoacidosis (DKA) and to ascertain whether breath acetone can be used in monitoring and controlling the disease. The high amount of ketone bodies in the blood is usually an indicator that the catabolism of fatty acid is greater than the one for carbohydrates [29,30]. In this study, 30 diabetic and 28 non-diabetic patients’ fasting serums were analyzed for ketone bodies (acetoacetate and beta-hydroxybutyrate) using the Abcam^®^ acetoacetate and beta-hydroxybutyrate assay kits (Figure 3). Other clinical data, which include blood glucose, glycated hemoglobin, total cholesterol, triglycerides, high-density lipoprotein and low-density lipoprotein were also measured to confirm the state of diabetes mellitus (Table 1).

Breath acetone from both diabetic and non-diabetic patients was analyzed with the HS-SPME/GC-MS. As expected, guided by literature, a high amount of breath acetone was observed in diabetic patients (more than 0.8 ppm), as opposed to their non-diabetic (less than 0.8 ppm) counterparts. The mean values of acetoacetate in diabetic and non-diabetic patients were 0.09 mmol/L and 0.05 mmol/L, respectively. The mean β-hydroxybutyrate was also higher in diabetes patients, 0.46 mmol/L), as compared to the non-diabetes patients 0.25 mmol/L. As shown in Table 1, plasma glucose and total cholesterol were slightly higher in type 2 diabetes mellitus than non-diabetes mellitus. Triglycerides, HDL cholesterol, LDL cholesterol, β-hydroxybutyrate and acetoacetate levels were not significantly different between the two groups.

### 3.2. Breath Acetone Analysis Using HS-SPME/GC-MS

**Figure 4:**
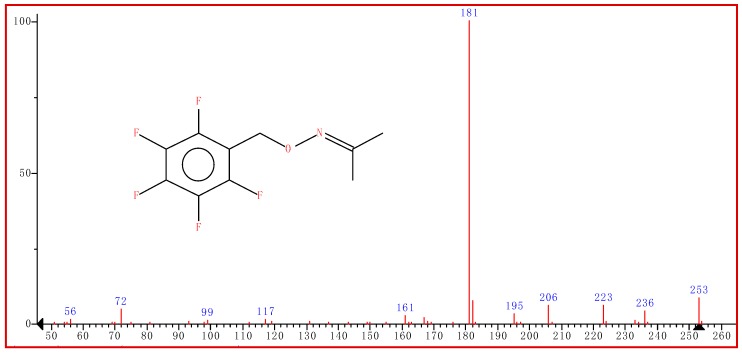
The gas chromatography-mass spectrometry (GC-MS) mass spectrum of acetone-oxime.

In this study, HS-SPME/GC-MS analysis was applied to determine the concentration of acetone in the breath of 30 diabetic and 28 non-diabetic mellitus patients. Given the small mass of acetone (58 amu) and its volatility, the acetone in breath was first derivatized with a derivatizing reagent, pentafluorobenzyl-hydroxylamine-hydrochloride (PFBHA) prior to the GC-MS analysis. The reaction of acetone in the breath with a derivatizing agent (PFBHA) forms a very stable acetone-oxime, and Figure 4 shows the mass spectrum of the acetone oxime with a base peak at *m/z* 181 which was extracted for quantitative purposes. The amount of oxime formed on the fiber is proportional to the acetone concentration in the breath. Acetone concentration higher than 1.8 ppm was observed in some of the diabetic breath (Figure 3a) and acetone concentration lower than 0.8 ppm was observed (Figure 3b). The GC spectrum of both diabetic and non-diabetic mellitus volunteers are available on the supplementarary results in Figure S1.

Determination of acetone in breath using HS-SPME/GC-MS with on-fiber derivatization yielded satisfactory precision and an excellent sensitivity with a simple procedure. The present method is a potential tool for a non-invasive diagnosis and monitoring of diabetes mellitus and ketoacidosis.

### 3.3. Correlation Studies of Breath Acetone and Blood Ketone Bodies

We observed the correlation coefficient of *r* = 0.897 between breath acetone and plasma acetoacetate, and we further observed a good correlation of *r* = 0.821 between breath acetone and plasma beta-hydroxybutyrate. This shows a positive indicator of using acetone as a non-invasive biomarker of diabetes mellitus. The blood glucose and acetone also demonstrated a good correlation. The results are found in the Supplementary Material, Figure S2.

## 4. Discussion

To our knowledge, this study demonstrates for the first time that blood ketone bodies correlate very well with breath acetone in type 2 diabetes mellitus patients. Many studies have reported on either the correlation or analysis of ketone bodies in the blood and breath of type 1 diabetes mellitus, but have never reported ketone bodies on type 2 diabetes mellitus patients. Generally, blood glucose, acetoacetate, beta-hydroxybutyrate and acetone levels differ from individual to individual, and it also varies from day to day, even for the same individual, as can be seen in Figure 3. It depends on the everyday diet, medications, stress and physical activities [31]. In this study, different diabetic and non-diabetic mellitus patients showed the above-mentioned characteristics, thus all the non-diabetic patients showed different plasma metabolites levels. The plasma glucose mean value of 8.55 mmol/L in diabetic and 5.72 mmol/L in non-diabetic patients were observed.

Some diabetic patients showed very good plasma glucose, total cholesterol, triglycerides, HDL cholesterol and LDL cholesterol levels even if they were diagnosed with diabetes mellitus. Thus these patients control their diet, medication and exercise well. Whilst in other diabetic patients, uncontrolled plasma blood glucose levels (11.30, 28.30, 10.30, 10.80, 10.00 mmol/L) were observed. Some patients showed very low plasma glucose levels (3.90 mmol/L) which indicates the state of hypoglycemia that is usually observed in type 1 diabetes mellitus patients. The plasma glucose confirms that the patients are diabetic, and some patients can monitor their glucose level.

Checking for blood glucose alone does not give a clear state of diabetic danger. Hyperketonemia in diabetes is due to insufficient insulin action. It has also been observed in other endocrine-related diseases where excess hormone secretion antagonizes insulin action [14]. Using the Abcam^®^ acetoacetate and beta-hydroxybutyrate assay kits on 30 diabetes and 28 non-diabetes patients, we observed a physiological amount of ketones bodies with the mean values of 0.09 and 0.46, respectively, for diabetes, and 0.05 and 0.44, respectively, for non-diabetes patients. Furthermore, beta-hydroxybutyrate and acetoacetate concentration might provide more information about the severity of ketoacidosis, whether it is related to diabetes, alcohol, or starvation [32]. A blood ketone level less than 0.5 mmol/L is considered to be physiological, whereas hyperketonemia is defined by a value greater than 1 mmol/L, and ketoacidosis is considered to be probable above 3 mmol/L [31,33]. We did not observe hyperketonemia in this study group. However, this does not mean that type 2 diabetes does not undergo hyperketonemia, but simply implies that the patients are able to control their disease. It has been reported that ketone bodies were higher in insulin-dependent patients than non-insulin dependent patients. However, they have found a good correlation of ketone bodies and skin acetone even in controlled diabetes. Our study also found a good correlation in some controlled type 2 diabetes.

Quantifying breath acetone is of importance to this study, as we hope to find the significant correlation between plasma ketone and breathe acetone. Thus, it will strengthen the movement of finding a portable chemoresistive acetone sensor device that will be able to detect acetone from the human breath from as little as 0.1 ppmv. While other non-invasive methods of detection exist such as urine, breath is a less complicated mixture than urine in a sense that it is amenable to complete the analysis of all compounds present. Thus no workup of breath samples are required, in contrast to many analyses performed on urine samples. Additionally, it provides direct information on the respiratory function that is not obtainable by other means. Using HS-SPME/GC-MS, we successfully quantified the acetone level in the breath of both diabetic and non-diabetic mellitus patients. The reaction of acetone in the breath with a derivatizing agent (PFBHA) forms very stable acetone-oxime that was presented on the mass spectrum of the acetone oxime with a base peak at *m*/*z* 181. Acetone concentration higher than 1.8 ppmv was found in diabetic breath (Figure 3a). For non-diabetic breath, acetone concentrations lower than 0.8 ppmv were observed (Figure 3b), and the GC-MS spectrum is within the Supplementary results. This study is consistent with the literature [9,14,32,33,34].

**Figure 5:**
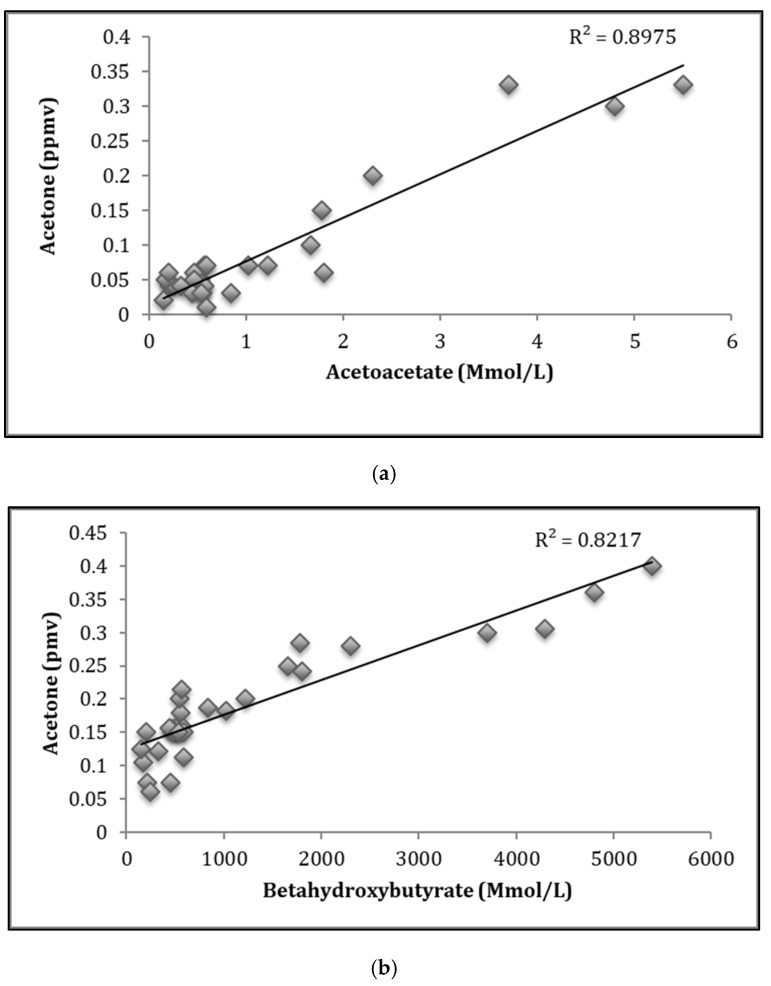
(**a**) Correlation between breath acetone and acetoacetate; (**b**) Correlation between breath acetone and beta-hydroxybutyrates. The correlations were calculated using linear regression.

After successfully determining the plasma concentration of acetoacetate, β-hydroxybutyrate and breath acetone, it was necessary to check the correlation of the blood ketones with breath acetone. The diversity of ketone bodies among 30 diabetes patients appeared at baseline (Figure 5). Significant positive correlations between breath acetone and blood AcAc and between breath acetone and blood β-OHB were observed at baseline (*R* = 0.897 and *R* = 0.821). This shows a positive indicator of using acetone as a non-invasive biomarker of diabetes mellitus. There are many hypotheses to explain the relationship. One reason being that acetone is a metabolite produced after enzymatic decarboxylation of AcAc, which is in equilibrium with β-OHB via an enzymatic-controlled process by β-OHB dehydrogenase [9]. Although an exponential relationship between acetone and β-OHB, and acetone and AcAc, were observed, acetone reflected overall ketone metabolite concentrations in diabetic patients. This is due to the fact that acetone presents positive deviations from well-known gas/liquid partition laws, such as Henry’s law or Raoul’s law.

## 5. Conclusions

The HS-SPME/GC-MS was used to successfully quantify the amount of breath acetone in type 2 diabetes mellitus and non-diabetes patients. Blood glucose and ketone bodies were also measured. The high amount of ketone bodies (acetone, acetoacetate and beta-hydroxybutyrate) were observed in diabetic patients as opposed to non-diabetic mellitus patients. Breath acetone levels were found to increase with blood β-hydroxybutyrate and blood acetoacetate levels. This might suggest a potential to develop breath gas analysis diagnostic tools to provide an alternative to blood testing for both type 1 and type 2 diabetes mellitus monitoring, and to assist with the prevention of diabetic ketoacidosis.

## Figures and Tables

**Table 1:** Clinical data of type 2 diabetes mellitus (DM) and non-diabetes mellitus.

Biochemical Parameters	Type 2 DM (*n* = 30)	Non-Diabetes (*n* = 30)	*p*-Value
Age	47 ± 10	41 ± 10	<0.001
Gender	13/17	11/19	0.10
BMI (kg·m^−2^)	28.4 ± 4.5	25.4 ± 4.0	0.47
Plasma glucose (mmol/L)	8.6 ± 2.43	5.7 ± 1.44	0.007
HB1Ac (%)	10.3 ± 2.57	-	-
Total cholesterol (mmol/L)	5.10 ± 1.40	4.5 ± 1.42	0.17
Triglycerides (mmol/L)	1.57 ± 1.3	1.04 ± 1	0.01
HDL cholesterol (mmol/L)	1.15 ± 0.27	1.33 ± 0.47	0.34
LDL cholesterol (mmol/L)	2.56 ± 1.32	2.43 ± 0.97	0.52
Β-hydroxybutyrate (mmol/L)	0.46 ± 0.02	0.44 ± 0.41	0.55
Acetoacetate (mmol/L)	0.09 ± 0.02	0.05 ± 0.03	0.47

Data in mean ± standard deviation (SD), BMI (body mass index), HDL (high-density lipoprotein) and LDL (low-density lipoprotein).

## Supplementary Materials

The following are available online at https://www.mdpi.com/2075-4418/9/4/224/s1, Figure S1: Reconstructed GC-MS ion chromatograms (*m/z* 181) of patient breath samples without insulin injection (a), diabetic breath with insulin (b), and non-diabetic breath (c) sampled using on-fiber SPME derivatization with PFBHA. Figure S2: The measured breath acetone concentration by SPME GC/MS and versus blood glucose in diabetic patients.
