# Supplementary material for: Blood Ketone Bodies and Breath Acetone Analysis and Their Correlations in Type 2 Diabetes Mellitus
Source: Diagnostics (Basel). 2019 Dec 17;9(4):224. doi: 10.3390/diagnostics9040224 (PMC6963753; doi:10.3390/diagnostics9040224)
Supplement: Supplementary file 1 [file diagnostics-09-00224-s001.pdf]

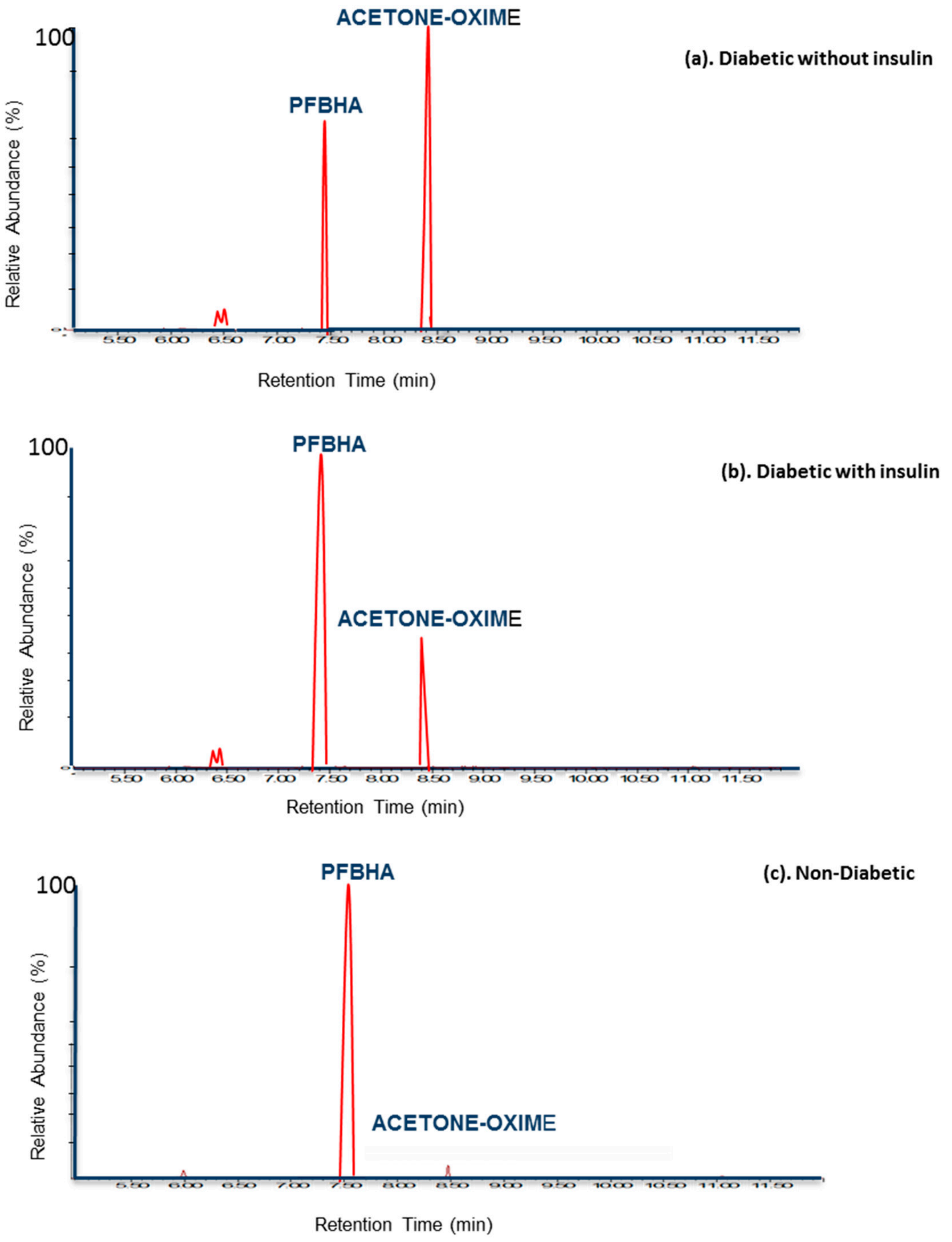

Figure S1. Reconstructed GC-MS ion chromatograms (m/z 181) of patient breath samples without insulin injection (a), diabetic breath with insulin (b), and non-diabetic breath (c) sampled using on-fibre SPME derivatization with PFBHA.

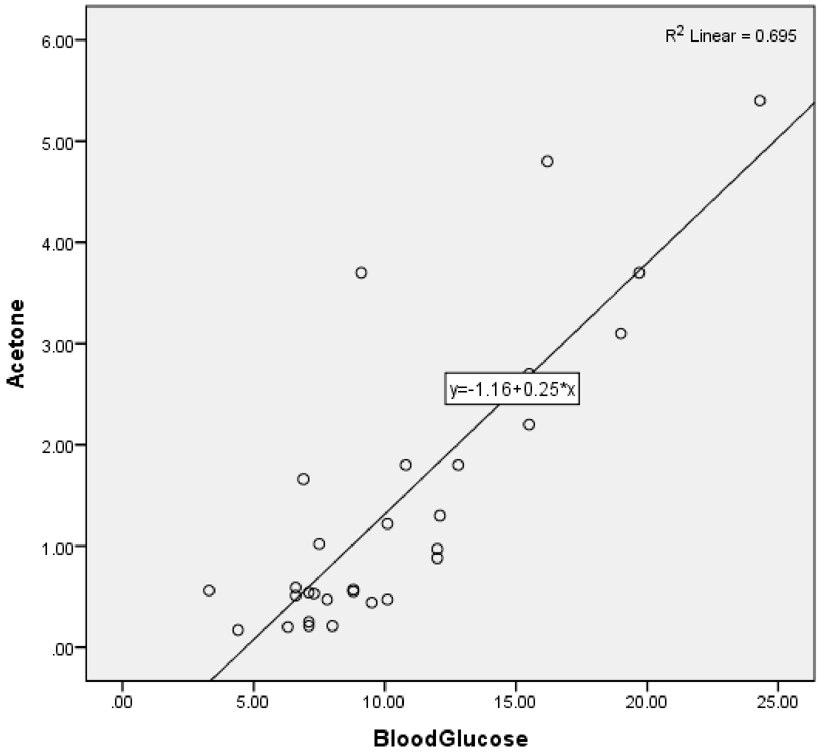

Figure S2: The measured breath acetone concentration by SPME GC/MS and versus blood glucose in diabetic patients.
